# Quality assessment and community detection methods for anonymized mobility data in the Italian Covid context

**DOI:** 10.1038/s41598-024-54878-0

**Published:** 2024-02-26

**Authors:** Jules Morand, Shoichi Yip, Yannis Velegrakis, Gianluca Lattanzi, Raffaello Potestio, Luca Tubiana

**Affiliations:** 1 University of Trento, via Sommarive 14, 38123 Trento, Italy; 2 INFN-TIFPA, Trento Institute for Fundamental Physics and Applications, 38123 Trento, Italy; 3 Utrecht University, Princetonplein 5, 3584 CC Utrecht, The Netherlands

**Keywords:** Information theory and computation, Complex networks, Statistical physics, Applied mathematics, Scientific data, Statistics

## Abstract

We discuss how to assess the reliability of partial, anonymized mobility data and compare two different methods to identify spatial communities based on movements: Greedy Modularity Clustering (GMC) and the novel Critical Variable Selection (CVS). These capture different aspects of mobility: direct population fluxes (GMC) and the probability for individuals to move between two nodes (CVS). As a test case, we consider movements of Italians before and during the SARS-Cov2 pandemic, using Facebook users’ data and publicly available information from the Italian National Institute of Statistics (Istat) to construct daily mobility networks at the interprovincial level. Using the Perron-Frobenius (PF) theorem, we show how the mean stochastic network has a stationary population density state comparable with data from Istat, and how this ceases to be the case if even a moderate amount of pruning is applied to the network. We then identify the first two national lockdowns through temporal clustering of the mobility networks, define two representative graphs for the lockdown and non-lockdown conditions and perform optimal spatial community identification on both graphs using the GMC and CVS approaches. Despite the fundamental differences in the methods, the variation of information (VI) between them assesses that they return similar partitions of the Italian provincial networks in both situations. The information provided can be used to inform policy, for example, to define an optimal scale for lockdown measures. Our approach is general and can be applied to other countries or geographical scales.

Diffusion processes in human society depend on the complex structure of the underlying network of interactions. In principle, these can be studied at the individual scale, where each node corresponds to an agent, for example through social experiments recording the contacts of a group of people *via* special devices. This was done e.g. in a summer camp for children in Italy^1^ or with primary and high-school students in France^2–4^. Such data can then be used to generate a time-dependent network of contacts that can be later used to simulate the diffusion of an epidemic and study how it propagates at the scale of individuals^5,6^. At larger scales, privacy concerns and pragmatic necessities make it preferable to turn towards the use of aggregated data and meta-population network models^7,8^, where nodes represent groups of people, administrative territories or States. This can be done for example at a national^9^ or international level ^10,11^, or at multiple levels through the usage of multi-scale information on people’s mobility^12^. Meta-population models are often informed by anonymized data such as airplane traffic^10,11^ or social network location data^13^. The contact networks can also be inferred from the infectious process through a Bayesian approach^14,15^. The typical approach in these studies consists in mapping interactions and circulations onto time-series of weighted directed graphs and in finding relevant patterns in such complex, dynamical, networks.

Identifying mobility patterns can be particularly important in the case of an epidemic, such as the recent SARS-CoV-2 pandemic^16^, as they provide valuable information to model its spreading. To minimize the impact of an epidemic governments must take far-reaching decisions with large impacts on the lives of their citizens. Some prevalent measures deployed during the pandemic to contain it were the adoption of personal protection devices such as face masks^17,18^ or contact tracing aimed at identifying and confining infectious subjects^19–24^. Yet, the most common measure adopted and experienced across the world was the use of various forms of general lockdown to dampen the large-scale contagion^25–30^.

While lockdowns are certainly effective in reducing people's mobility, as proven by several studies^31–34^, and thus curbing the rise of infections, their imposition severely affects the lives and health of citizens^35–37^. The extent of their deployment thus needs to be optimized both in space and time to minimize the number of people affected, while guaranteeing the safety of the population. Mobility patterns obtained through spatial community identification can provide useful insights in this direction. It is then crucial to take advantage of a data-driven approach to suggest the most relevant scale for partitioning of a territory. Here, we show how this optimal scale can be captured through a temporal and spatial clustering approach on a data-driven meta-population model based on anonymized aggregated data.

As a case study, we analyze the mobility network of the Italian population during the COVID-19 crisis between January 2020 and May 2022: a period including large and diverse variations in mobility fluxes. Italy was the first European country to impose a national lockdown and has seen the implementation of three nationwide lockdowns: between March and April 2020, in January 2021, and in April 2021. Detailed studies have been carried out on the initial propagation of the epidemic in Italy^38,39^, on the first confinement^40^ and its relaxation^41^, discussing the necessity and the implementation of such restrictive measures. After the first phase of the pandemic, the Italian government delegated part of the responsibility of restrictions to regional governments, which were forced to curb the movements of their citizens whenever the effective reproduction number $R_t$ (i.e. the average number of new infections caused by a single infected individual at time *t*) went above 1^42–44^. Imposing regional lockdowns instead of national ones is a sensible strategy. However, it is not guaranteed that existing administrative regions correspond to the best subdivisions of a state to control the spread of epidemics. Statistical approaches, like community clustering, can be used to analyze mobility data in order to identify the best areas or macroregions that should be monitored together. The results obtained by community identification algorithms depend on the quality of the available –in general partial and anonymized – data, on the adopted algorithm and its parameters, and on the observable being optimised. In this study, we propose a pipeline to assess the robustness of partial and anonymized mobility data by leveraging the Perron-Frobenius theorem for stochastic matrices and identify spatial communities using two different methods: the recently introduced Critical Variable Selection (CVS)^45^ scheme, based on an information-theoretical optimization, and the Greedy Modularity Clustering (GMC), based on graph theory.

GMC, based on modularity (a quantity computed from the degree of the nodes) only account for information coming from the first neighbors in the network, while CVS is based on a distance matrix between any pair of nodes. Furthermore, in our approach we base GMC on the fluxes of people moving between provinces, and CVS on a distance matrix that captures the probability for an individual to travel between any two provinces, accounting for all possible trajectories between them.

The proposed method is generic to any temporal weighted directed graph and can be applied to other countries, at different geographical scales, and also to similar networks (e.g. biological networks) to find temporal features and to cluster their nodes and their complexity.

To test our approach we consider the case of Italy between February 2020 and May 2022 and estimate the mobility network of the Italian population at the level of provinces (small administrative regions between municipalities and regions) thanks to Facebook (FB) data obtained through META’s *Data for Good* program^46^. While these datasets are perhaps too sparse to be used in the analysis and simulation of detailed epidemic scenarios^47^, due also to excessive pruning^48^, they are still sufficient for our analysis, as the resulting networks are strongly connected. Furthermore, our approach allows one to get at least a qualitative idea of the impact pruning has had on the mobility networks, by comparing the stationary population density vector corresponding to a stochastic process based on the average mobility network with the density vector obtained through third-party data. In this study, we used as a reference the data from the official projected census for January 1st, 2020^49^ from the Italian National Institute of Statistics (Istat^50^).

The manuscript is organized as follows. In section “Transition matrices” we define the averaged daily mobility matrices. In section “Homogeneity and representativeness of FB data” we discuss data validation, show that the average population density obtained from the FB data is in good agreement with the one from Istat and how pruning severely affects this agreement. In section “Temporal clustering” we perform a temporal clustering of the mobility matrices to identify the lockdown periods. This allows us both to perform a second check on the quality of the data and to define too representative matrices for these two periods to be used to perform the spatial clustering. This is done in section “Optimal spatial clustering” where we compare the results from community-clustering and CVS. An analysis based on variation of information shows that the two methodologies are in good agreement. The details of the algorithms and data are reported in Materials and Methods.

## Results

Our approach to characterize the behavior of the Italian population is based on movement data between provinces. These are administrative entities in between regions and municipalities, usually containing between one and three hundred thousand people, with those corresponding to major cities such as Rome, Naples, Milan, Turin, and Palermo having more than a million inhabitants^50^.

As explained in detail in the Methods section Datasets , we consider 106 provinces (see Table of Appendix A.) and extrapolate the movement of their respective populations from FB users’ data provided by META’s data for good program^46^. The dataset we used provides the number of FB users in each province *i*, $n_i$, as well as the number of users moving between two provinces (or within a province), $n_{ij}(t)$, every 8 h in the period between January 2020 and May 2022. More details about the data and their treatment can be found in SI Appendix I.

### Transition matrices

**Figure 1:**
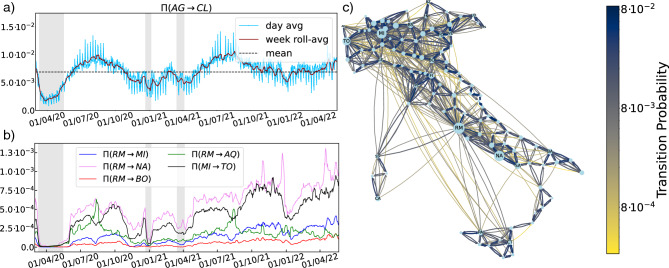
(**a**) AG$\rightarrow$CL (Agrigento to Caltanissetta provinces) link vs time. Daily average probability (blue) and 7-day rolled-average probability (red), and overall probability averaged in time (black dashed line). (**b**) Examples of some representative weekly rolled-average transition probability links. (**c**) Representation of the directed graph defined by the Matrix $\overline{\Pi }$ (Eq. 4). Arrows represent the mean probability links, $\overline{\Pi }_{ij}$, between Italian provinces *i* and *j*, and are scaled in size and color according to the value of the link (from gold to dark blue). Self-links $\Pi _{ii}$ are not shown. The size of the nodes is proportional to the population (vector $\varvec{\rho }^*$ of Fig. 2b).

The data from META allow us to compute the 8-h transition rate between two provinces *i* and *j*, defined as follows:

1 $$\begin{aligned} \Pi _{ij} (t) = \frac{n_{ij}(t)}{\sum _{j}n_{ij}(t)}. \end{aligned}$$

Note that the denominator ensures that, for every province *i*, $\sum _{j} \Pi _{ij} = 1$, thereby guaranteeing that $\Pi$ can be used as a stochastic matrix. To remove seasonal fluctuations in $\Pi$ (day vs. night, weekdays vs. weekends) we redefine $\Pi$ as the daily transition rate between provinces averaged over the 3 days before and 3 days after, see Materials and Methods section “Stochastic transition matrices”. This gives us weekly-averaged daily transition matrices. Finally, we also make use of the *mean transition matrix* over the whole period, $\overline{\Pi }$.

To get an idea of what the data look like, the time evolution of one link $\Pi _{ij}$, reporting the mobility from the province of Agrigento ($i=AG$) to that of Caltanissetta ($j=CL$), is plotted in Fig. 1a. Daily averaged values are reported in blue, weekly averaged ones in red, and the corresponding entry in the mean transition matrix $\overline{\Pi }$ in a black dashed line. The lockdown periods are indicated by grey-shaded vertical bars. Seasonal effects are clearly visible from the comparison of the daily data and the corresponding weekly averaged ones. A subset of weekly-averaged daily transition rates between different provinces is reported in Fig. 1b. The directed graph associated with $\overline{\Pi }$ is displayed in Fig. 1c.

**Figure 2:**
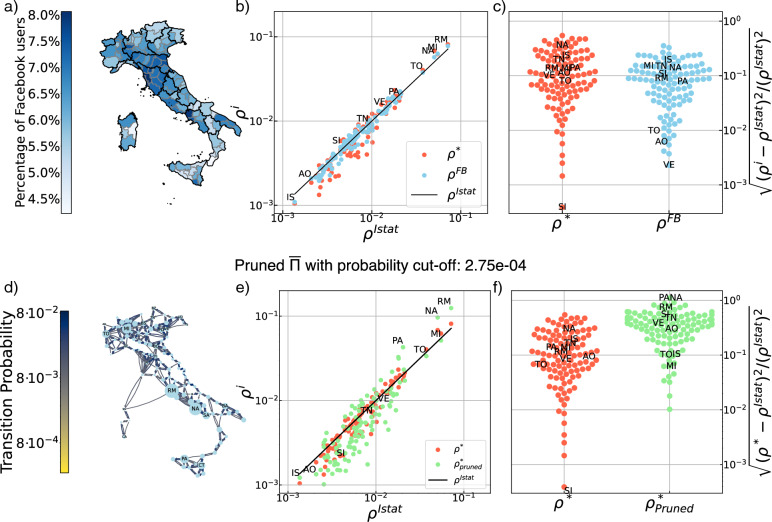
(**a**) Fraction of FB users that have shared their location over the official province population obtained from the Istat 2020 census, $\overline{n_i}/n_i^{\text {Istat}}$, for each province *i*. (**b**) Comparison of the different population density vectors from FB and Istat data: $\varvec{\rho }^{\text {FB}}$ and $\varvec{\rho }^{*}$ are plotted against $\varvec{\rho }^{\text {Istat}}$. (**c**) Standard deviation of the vectors $\varvec{\rho }^{\text {FB}}$ and $\varvec{\rho }^*$ from the $\varvec{\rho }^{\text {Istat}}$ vector. (**d**) Representation of the directed mobility graph without links smaller than a cutoff of probability $2.75\cdot 10^{-4}$, normalised so that the corresponding pruned matrix is a stochastic matrix. The size of the nodes is proportional to the Perron-Fobenius first left eigenvector, $\varvec{\rho }^*_{pruned}$, of the pruned matrix. (**e**) $\varvec{\rho }_{pruned}^*$ together with $\varvec{\rho }^*$ versus $\varvec{\rho }^{Istat}$ (**f**) The standard deviation of $\varvec{\rho }^*$ and $\varvec{\rho }_{pruned}^*$ from $\varvec{\rho }^{Istat}$. With higher values of the cutoff the graph becomes only weakly connected and the assumptions for PF break. (see also Fig. SI.2. Appendix C.).

### Homogeneity and representativeness of FB data

We assume that the FB users in the database are homogeneously distributed across provinces, and move in a manner that is on average similar to that of the rest of the population. To validate these assumptions we proceed as follows.

First, we monitor the fraction of FB users over the total population of the province according to Istat; this ratio is defined as $\overline{n_i}/n_i^{\text {Istat}}$, where $\overline{n_i}=\left<n^h_i\right>$ is the number of FB users in province *i* averaged over the whole time series. The results, reported in Fig. 2a, show that in all provinces this fraction remains between $3\%$ and $7\%$, and that FB users are roughly homogeneously distributed across the country.

A more quantitative validation of both assumptions can be obtained by considering the *population density vectors* obtained both from the official census of Istat in 2020 and from FB users’ data. These are defined as follows:

2 $$\begin{aligned} \varvec{\rho }=\left( \frac{n_{1}}{n_{tot}},\ldots ,\frac{n_{N}}{n_{tot}}\right) ^{T} \text {where }n_1, \ldots n_{N}\; \text {are the populations of the }N\; \text{provinces, and } n_{tot} = \sum _{i=1}^{N} n_{i}\text { is the total population.} \end{aligned}$$

The populations $n_{i}$ can be obtained from either: Istat data, $\varvec{\rho }^{\text {Istat}}$ or the FB population dataset $\varvec{\rho }^{\text {FB}}$. The above normalization, Eq. (2), sets $|\varvec{\rho }|=1$ and allows us to compare the different vectors. In addition, it is possible to compare another population density vector, $\varvec{\rho }^*$, obtained from the mean matrix $\overline{\Pi }$ extracted from the FB movement dataset.

In the graph described by $\overline{\Pi }$ there is a non-zero probability to reach any node from any other one in a finite number of steps, that is, the graph is strongly connected and aperiodic, and random walks over it are ergodic. The Perron-Frobenius (PF) theorem then ensures that $\overline{\Pi }$ has a non-degenerate highest eigenvalue. With our normalisation of $\overline{\Pi }$ this is $\lambda ^*=1$, and its associated left eigenvalue $\mathbf {\rho }^*$ is the only stationary state of the system, satisfying: $\rho ^*_i \overline{\Pi }_{ij}= \overline{\Pi }_{ji} \rho ^*_j.$

Therefore, any non-trivial distribution vector over the nodes of our network will converge to $\varvec{\rho }^*$ after a sufficiently long time (see SI Appendix C). If the movements described by $\overline{\Pi }$ are consistent with the Istat population data, the stationary density vector $\varvec{\rho }^*$ must be in good agreement with the Istat density vector $\varvec{\rho }^{\text {Istat}}$. This is indeed the case, as shown in Fig. 2b,c.

Figure 2, panel b) displays the population density vectors $\varvec{\rho }^{\text {FB}}$ and $\varvec{\rho }^{*}$, on a log-log scale against $\varvec{\rho }^{\text {Istat}}$. The provinces are sorted from least to most populated according to Istat data. We see a good agreement within the FB data themselves, which is also a benchmark of our extraction and preparation of the data.

Moreover, the standard deviations of $\varvec{\rho }^{\text {FB}}$ and $\varvec{\rho }^{*}$ from the Istat vector (panel c) of Fig. 2) are in very good quantitative agreement with the Istat data. However, we notice that the most populated provinces, Rome, Milan, Naples, and Turin, (RM, MI, NA, TO) are slightly overestimated and that the less populated provinces are slightly underestimated especially by the $\rho ^*$ vector. This can be explained by the fact that all links with less than 10 people are ignored for privacy reasons.

In the last row of Fig. 2, panels d), e), f) show the validity of the method: using a pruned mean mobility matrix, we see its stationary PF vector deviating more from the National data. The pruning consists in removing all links of the mean matrix corresponding to transition probabilities below $2,75.10^{-4}$, as shown on the graph representation on panel d). The pruned matrix is then normalised to be stochastic, and the PF stationary vector, $\varvec{\rho }_{pruned}^{*}$, is computed. We see it on panel e) compared to $\varvec{\rho }^*$ and $\varvec{\rho }^{Istat}$. In panel f) we compare the standard deviation of the two PF vectors with respect to the Istat one. We see a clear deviation of the PF vector from the Istat vector when using the pruned (less detailed) matrix. The PF method completely breaks off if the graph is no longer strongly connected. This result is presented in Appendix C of the SI, Fig. SI.2, where we report the results from a progressive pruning up to the breaking point. It is interesting to note not only the increasing deviation from the Istat data but also how some nodes, not necessarily the most or least populated ones, become large sinks or sources of the diffusive process.

Finally, we note that a weaker check can be done internally, using the vector $\rho _{FB}$ provided by META and comparing it against the stationary population density vector $\rho ^*$. Increasing the pruning, the difference between the two populations will increase.

Having validated the FB data, we can proceed to extract the information contained in the time series of weekly-averaged daily transition matrices. First of all, we notice that diagonal elements $\Pi _{ii}\ge 0.9$, meaning that most movements happen within provinces. Second, and most notably, we find that while the time series of the probability to move between different provinces can vary by an order of magnitude, as shown in Fig. 1b, the movement pattern of single provinces can be brought to collapse on two master curves with an appropriate re-scaling, see SI, Appendix D and E, and in particular Figs. SI.4 and SI.5.

### Temporal clustering

In order to use movement data to identify spatial communities, we first need to identify the confined and unconfined periods, as the mobility was considerably reduced during lockdown periods compared to the rest of our 2-year time window. This also provides a further quality check for the data contained in the transition matrices $\Pi (t)$.

**Figure 3:**
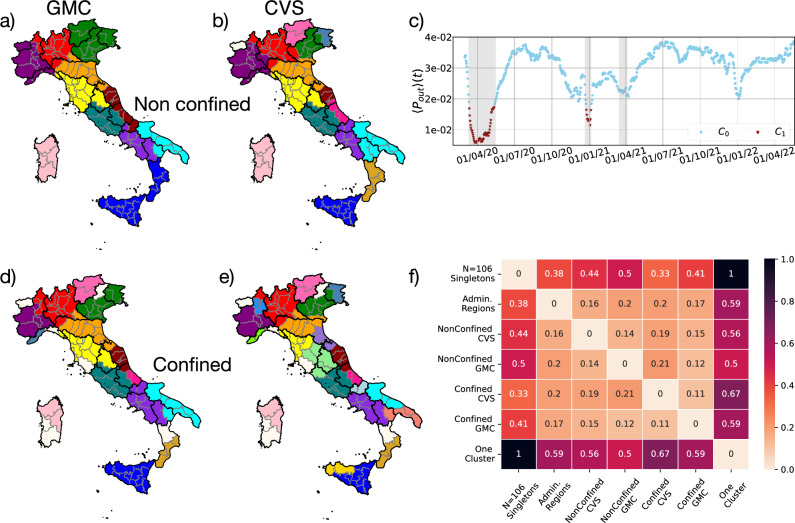
Left panels: (**a**,**d**) Spatial community clustering obtained with Greedy Modularity and, (**b**,**e**) Critical Variable Selection. Panels (**a**), (**b**) report the communities identified by the two methods during the non-confined periods. Panels (**d**), (**e**), during confinement. Grey lines represent the borders of the provinces while bold black lines delimit administrative regions. Right panels: (**c**) Temporal clustering: Mean mobility $\langle P_{out}\rangle (t)$ versus time. The light blue dots and dark red stars illustrate the two temporal clusters of transition matrices series. Gray-shaded areas represent national confinement periods. (**f**) Variation of Information VI between the different partitions of Italy presented above, VI is here divided by $\log (N)$ to provide a 0 to 1 scale.

To do identify the lockdowns, we cluster the daily movement matrices into two groups based on the distance induced by the matrix-matrix scalar product, as described in the Materials and Methods section “Temporal clustering method”. The results are reported in Fig. 3c, where each matrix is represented by the average probability for people to move out of their province at time *t*:

3 $$\begin{aligned} \left<P_{out}\right>(t)=\frac{1}{N} \sum _{i=1}^N 1-\Pi _{ii}(t)= 1-\frac{1}{N}{{\,\textrm{Tr}\,}}(\Pi (t)). \end{aligned}$$

The two temporal clusters $C_0$ and $C_1$ are represented by light blue dots and dark red stars, respectively, and the latter clearly identifies the first two national lockdown periods, delimited by the vertical shaded areas. Although the third lockdown period is not identified by the clustering, we argue that this is because it has not been strictly imposed, nor was it effectively respected, as it can also be seen from the mobility plots of Figs. 3c and SI.2.

FB data thus entails mobility features that are in agreement with the history of the Italian government’s decisions and their repercussions on the population’s behaviour, validating their usage in modeling epidemics and social phenomena more in general.

### Optimal spatial clustering

We can now perform a spatial clustering of the most representative matrices of the two temporal clusters obtained for the confined and unconfined situations.

To this aim, we define for each of the two temporal clusters ($C_k, \, k=0,1$):

- The *mean transition matrices* $\overline{\Pi }^{C_k}$ ,
- The *most representative transition matrices* $\widetilde{\Pi }^{C_k}$,
- The *most representative current matrices* $J^{C_k}\!\!=\widetilde{\Pi }^{C_k}\rho ^{\text {Istat}}$.

We then use two different methods to perform and optimise the clustering:

- The Greedy Modularity Communities method uses the flux of people moving between nodes, $J^{C_k}$. This corresponds to the probability for a randomly picked Italian to be in a province, $\rho ^{Istat}$ multiplied by the probability of moving. This approach maximises the *modularity* (section Greedy modularity communities method (GMC)) of a clustering, resulting in partitions whose clusters have higher fluxes within themselves than between different clusters. It is important to note that modularity only uses information about nodes directly connected by an edge (first neighbors).
- The Critical Variable Selection method is based on a distance matrix between any pair of nodes in the network. This includes pairs that are not directly connected. This matrix is computed starting from the transition probability $\widetilde{\Pi }^{C_k}$. Each entry of $\widetilde{\Pi }^{C_k}$ gives the probability for a person picked in a node *A* to travel to any neighbor node *B*, without multiplying it by the population density of *A*. $\widetilde{\Pi }^{C_k}$ is transformed into a distance matrix by taking into account all possible paths leading from any node *A* to any node *B*, including those paths that traverse other nodes^51^ (Section Effective distance matrix between nodes). The optimal clustering maximizes the *relevance*, a quantity introduced in information theory (Section Critical Variable Selection method). CVS identifies the partition that minimizes information loss with respect to a full description of the dataset^45^.

The details of both strategies are reported in the Materials and Methods section Spatial clustering and a graph representation of the most representative matrix in each case can be found in Figs. S8 and S9 of Appendix H of SI. We observe here that, although in principle geographically distant provinces could be grouped together (e.g. in the case of highly connected cities such as Rome, Naples, Milan, and Turin), the clusters found by both methods are composed of physically proximal provinces, which can be reached one from the another without having to cross other clusters. This is a non-trivial result, as neither method relies on the notion of geographical distance.

#### Non confined

Figure 3a,b represent the clustering of the most representative matrix of the unconfined temporal cluster ($C_0$ in blue in the top panel of Fig. 3c corresponding to an ‘ordinary’ Italian mobility situation; the top map is obtained employing the greedy modularity method, while the bottom one makes use of the CVS approach.

The two methods return slightly different partitions: for the greedy modularity (top), the Italian provinces are grouped in 11 clusters corresponding to well-defined geographical areas, while 16 groups are found using the CVS scheme. Apart from a few border cases, the clusterings seem to reproduce well some known cultural and commercial ‘blocks’ within the Country. For example, the green cluster corresponds to the *Triveneto* area (that is Veneto, Friuli-Venezia Giulia, and Trentino-Alto Adige), while Sardinian provinces are fully grouped in their own cluster. The time series of outward and inward probabilities for each province are also displayed in the supporting information (Fig. SI.5.) for each optimal spatial cluster obtained with the greedy modularity method. We further computed the mobility Z-score for each province and found it to correlate, at least qualitatively, with their touristic vocation, with more touristic provinces showing the highest Z-score, see Fig. SI.6.

#### Confined

Things change dramatically when the matrix representing the confined case, $C_1$: cluster 1, in red in Fig. 3c, is considered. Fig. 3d,e display the corresponding clustering, in the left panel using GMC and in the right one using CVS. In this case, the optimal clustering produces 23 spatial clusters with the former approach and 30 with the latter. Both of them predict more clusters, as expected when mobility is reduced. By analyzing the most representative matrices as directed graphs, one can also see that the one for the confined case presents fewer links than the one for non-confined mobility and that some provinces become singletons in the optimal spatial clustering, see supporting information Appendix H: Figs. SI.8 and SI.9.

#### Comparison between the partitions obtained from GMC and CVS

In both the unconfined and confined case, GMC and CVS provide comparable results, as can be seen from Fig. 3a,b,d,e, with mostly local changes. In particular, the isolated provinces in the confined case (off-white in Fig. 3d–e are the same according to both strategies.

We quantify the similarity between the partitions found through GMC and CVS by measuring the *Variation of Information* (VI) between them. This observable quantifies the amount of information needed to pass from one partition to another and has been adopted for example in the context of subfamily classification of protein in phylogenomics^52^. Importantly, VI defines a metric in the space of all possible partitions of a given set of N objects^53,54^. When normalized by dividing it by the logarithm of the number of elements in the set, it assesses how ‘close’, in terms of information, two partitions are on a scale from 0, for identical partitions to 1, the distance between a partition composed of N singletons and one including single cluster (the two extreme cases). We report its formal definition in Material and Methods 3.4.5.

Figure 3f shows the value of VI for the different partitions of Italy presented above. We normalize VI by dividing it by $\log (N)$ with $N=106$. On this scale, we see that $VI=0.14$ between the greedy community and CVS clustering during the non-confined period and $VI=0.11$ in the confined one. For comparison the *VI* between the 20 administrative regions and unclustered 106 provinces is 0.38, highlighting how seemingly small changes in *VI* can correspond to large reorganizations of the partition. It is then interesting to observe that the *VI* between the non-confined CVS/GMC clusterings and the unclustered provinces are both higher than 0.38. This is due to our clustering methods identifying fewer macroregions than the administrative Italian regions. The fact that the *VI* between the administrative regions and GMC is between 0.17 and 0.2 for confined and non-confined cases, while that between the administrative regions and the CVS is 0.2 and 0.16 is in agreement with the fact that our partitions identify macroregions that have large overlaps with the administrative ones, as seen in Fig. 3.

Looking at the local differences between the partitions identified by GMC and CVS can also provide useful information. We take as an example the unconfined clustering of Fig. 3a,b. The main differences are in the north-east (Trentino-Alto Adige, Veneto, Friuli-Venezia Giulia), Adriatic coast (Marche, Abruzzo, Molise), and the South (Calabria and Sicily). For each of these macroareas, GMC creates a single cluster, while CVS creates multiple smaller ones. To understand the origin of these discrepancies, we also computed GMC based on the transition matrix $\widetilde{\Pi }^{C_0}$, removing one of the different assumptions between the two methods. The result, reported in Fig. SI.10, shows that in this case GMC divides Friuli from Veneto and Calabria from Sicily like CVS, but keeps the Adriatic coast in a single cluster (in brown in Fig. 3a). By looking at the nodes connections reported in Fig. SI.10 the reasons becomes apparent. The nodes forming the Adriatic cluster are disposed on a single line. Modularity thus tends to cluster them together, while CVS tends to separate them into chunks of similar size.

## Conclusions

Picking the period 2020-2022 in Italy as a test case, we proposed a method to assess the quality of anonymized mobility data, identify spatial communities based both on Greedy Modularity Clustering and on the novel Critical Variable Selection method, and discussed how to extract information on the data based on their differences.

We showed how movement data from social networks (here META *Data for good* program) can be validated by considering the associated average transition matrix between nodes as the generator of a Markov jump process and comparing the corresponding stationary density vector with the population density vector obtained from the official census, or as an internal check with the population density available from the social network data itself. This criterion can in principle be extended to infer a corrected mobility matrix that reproduces the official census while remaining as close as possible to the starting one, leveraging an approach tested on in-silico generated gene-expression networks^55^. This could be particularly interesting as a way to infer the probability of small links in the mobility network. While these are in general not included in publicly-available mobility data in order to be compliant with privacy regulations, their absence can considerably reduce the usefulness of said data for modeling epidemic processes^47,48^. This problem will be tackled in a future study.

By considering the distance between transition matrices, we performed a temporal clustering to distinguish the lockdown periods from the rest. This successfully identifies the first two national lockdowns, which were strictly enforced by the Italian Government, and allowed us to define two representative mobility networks, one for the confined (lockdown) situation, and one for the unconfined case. We spatially clustered them according to GMC and CVS, comparing the results obtained from these methods. This comparison is important as GMC and CVS employ different algorithms and optimize different quantities (as detailed in 6.2). GMC is based on graph theory and identifies clusters so that the flux of people moving within them is higher than the flux between clusters. CVS identifies an optimal partition in terms of relevance, an information-theoretical quantity. Furthermore, GMC is limited to first-neighbor nodes, while CVS is based on a distance matrix defined between any pair of nodes in the network and based on all paths leading from one to the other.

Despite those fundamental differences, our results show that the two methodologies return comparable results, with only local variations, as captured by the VI measure. Analyzing these variations can provide further insights into mobility networks. In practice, the choice between GMC and CVS should be dictated by considerations on the kind of movements that one wants to cluster and the difference between the two clusterings highlights relevant differences in the identified communities, providing useful information to decision-makers.

Finally, we highlight that since our methodology is completely general, these strategies can be applied to other countries and other scales, as well as different problems relying on temporal varying networks. For example, identifying temporal and spatial clusters in the interaction networks between biomolecules is important to correlate their physical properties to their biological functions^56^, while clustering dynamic protein-protein interaction networks^57^ or gene regulation networks^58^ can provide relevant information on biochemical patterns within the cell as well as the co-regulation of genes, both of which are of fundamental interest for modern biological and pharmacological research. Dynamical networks are also extremely relevant in brain modeling, where the activation patterns between neurons are assumed to codify thoughts, memories, and reactions^59^. Mapping and understanding different patterns of activation is the focus of considerable research interest^60^.

## Materials and methods

### Datasets

#### Facebook movement data

The Facebook (FB) movement data were taken from META’s *Data for Good* program. The database records the number of people going from province *i* to province *j*, updated every 8 h, for Italian users who allowed FB to share such information with the app on their device; the time frame covered goes from March 1st, 2020 to May 22nd, 2022 (811 days). The database has been completely anonymized by META^61^. In particular, all links between two provinces containing less than 10 people are ignored.

The FB movement data are available both on a grid with cells of roughly $600 \times 600$ meters, which is the minimum tile size allowed for privacy protections (Bing tile level 16^62^), and at the scale of Italian provinces, administrative entities in between municipalities and regions. In this study we concentrate on the province level: the list of 106 provinces used was the official one in 2016 except for the provinces of Sud Sardinia (SU) and Cagliari (CA) which were merged into one node (CA), in order to get inter-compatibility of administrative regions between datasets from FB, Istat, and ISS. A map (Fig. SI.1 top right) and a table of these provinces can be found in the section “Result”. of supporting information. The appendix I of supporting information describes in detail the workflow of the data preparation.

In this database the FB data reports for each 8 h period (labeled by *h*):

- The number of FB users moving from province *i* to province *j* at time *h*, $n^h_{ij}$ (called $n_{crisis}$ in the original dataset).
- The total number of FB users in province *i* at time *h*, $n^h_i$.

### Istat and ISS data

The FB data cover only a fraction of the Italian population (namely those individuals who employ the FB app on mobile devices and have enabled their location sharing) and does not provide direct information on the population of each province, the amount of COVID cases registered there, nor the duration of confinement periods. The population of each province *i*, $n^{\text {Istat}}_i$, was obtained from Istat^50^, the Italian National Institute of Statistics. We used the most recent database available before the pandemic, released on January 1st, 2020. For simplicity, we assumed that the population remained constant during the period of study: this is an acceptable approximation, given that the global growth rate of the Italian population for that period is roughly $-0.4\%$^63^ and this fluctuation is negligible for our analysis.

The dates of the national confinements implemented by the Italian government are the following^44,64,65^: from 10/03/2020 to 16/05/2020; from 21/12/2020 to 06/01/2021; from 15/03/2021 to 05/04/2021.

The three periods are indicated by the grey-shaded areas in Figs. 1, 2 and 3. The confinement and de-confinement were progressive processes e.g. at first not all provinces were confined: only 2 days after the initial, local lockdown the measure was applied to the whole Country. Hence, we chose the temporal boundary of the lockdowns such that the periods correspond to the situation where the whole Country was confined, particularly periods in which any movement between provinces was prohibited. At smaller scales, national confinements were characterized by rigid restrictions on mobility^44^.

## Stochastic transition matrices

Using the data described in section “Facebook movement data”, we built the transition matrices between provinces. As described below, these are averaged daily and over the whole period.

## Mean transition matrix over the whole period

FB data allowed us to define a mean transition matrix $\overline{\Pi }$ between nodes as follows:

4 $$\begin{aligned} \overline{\Pi }_{ij}=\frac{\sum _h n^h_{ij}}{\sum _j\sum _h n^h_{ij}} \quad \text {where } \;\sum _h\text { is the sum over all 8-hour-slots during the whole data period.} \end{aligned}$$

The denominator in Eq. (4) normalizes the matrix such that the elements in each row sum to one: $\sum ^N_j \overline{\Pi }_{ij}=1, \forall i$, thus ensuring that $\overline{\Pi }$ is a stochastic matrix.

## Daily transition matrix

FB data were used to generate a daily transition matrix representing the link between provinces for each day, indexed by *t*. The time evolution of the mobility network was monitored by constructing a time series of transition matrices as follows:

5 $$\begin{aligned} \Pi _{ij}(t)=\frac{\sum _{h\in [t - \epsilon ,t+\delta ]} n^h_{ij}}{\sum _j\sum _{h\in [t - \epsilon ,t+\delta ]} n^h_{ij}} \quad \text {where } \; \sum _{h\in [t - \epsilon ,t+\delta ]}\text { is the sum over all 8-hour-slots in }[t - \epsilon ,t+\delta ]. \end{aligned}$$

Using Eq. (5) we constructed two different daily time series, one averaged every 24 h, $\epsilon =0$, and $\delta = 24$  h, and one based on a weekly rolling average, $\epsilon = 72$  h days, $\delta = 96$ h (in between 3 days before and 3 days after day *t*). The weekly averaged one correspond to the average of data provided by ISS.

### Temporal clustering method

To perform the temporal clustering of the transition matrices $\Pi (t)$, we used the standard Frobenius matrix distance between pair of matrices at time *t* and $t'>t$:

6 $$\begin{aligned} d(\Pi (t),\Pi (t'))=\sqrt{\sum ^N_{i,j}(\Pi _{ij}(t)- \Pi _{ij}(t') )^2}, \qquad \; t,t' \in \{ 1,\ldots ,T\} \end{aligned}$$

where *N* is the number of rows and columns in the transition matrices.

To identify the two clusters corresponding to confined (lockdown) and non-confined situations we applied a standard unstructured hierarchical clustering algorithm. In this bottom-up algorithm, the closest pairs of points and then pairs of clusters are recursively merged. We stop the algorithm when only two clusters are left. To find the closest clusters at each step, we compute the Frobenius norm between the mobility networks composing them and adopt the Ward linkage method, that is, we merge two clusters if the variance of the distance between the points in the resulting cluster is lower than the sum of the variances in the two original clusters. We implement this using the AgglomerativeClustering function available in the sklearn Python package^66^ version 1.2.2, specifying the target of two clusters, ward linkage, and an affinity matrix based on the Frobenius distance defined above. The other parameters are left to their default values.

### Spatial clustering

Spatial clusterings into communities are obtained starting from the most representative matrices of the two main temporal clusters $C_0$ and $C_1$; these correspond to the unconfined and confined periods respectively, and are represented in Fig. 3e.

### Most representative current matrices

We computed the mean matrices $\overline{\Pi }^{C_0}$ and $\overline{\Pi }^{C_1}$ of the matrices belonging to the unconfined ($C_0$) and confined ($C_1$) temporal clusters. From the mean transition matrices, we selected the most representative ones ($\widetilde{\Pi }^{C_k}$) of each cluster by taking the daily (weekly rolled-average) transition matrix closest to the mean and defined the most representative current matrix $J^{C_k}$ :

7 $$\begin{aligned} \widetilde{\Pi }^{C_k}=\min _{t\in {C_k}}{\Vert \Pi (t) - \overline{\Pi }^{C_k}\Vert },\, k\in \{0,1\}; \quad \text {and} \quad J^{C_k}_{ij}= \widetilde{\Pi }^{C_k}_{ij} \varvec{\rho }^{\text {Istat}}_i,\, k\in {0,1}, \quad \text {such that} \quad \sum ^N_{i,j} J^{C_k}_{ij}=1. \end{aligned}$$

where $C_k$ is the set of days ${t_i}$ within the temporal cluster *k*.

The transition matrices defined above provide the daily probability of going from one province to another, but the weights do not contain any information on the population of each province. To include this information, we constructed the current matrix by multiplying the most representative transition matrices of the two principal temporal clusters by the Istat vector $\varvec{\rho }^{\text {Istat}}$ and it is subject to the normalization condition of the most right equation above.

We specify here that we do not define the current matrix using the stationary (Perron-Frobenius) population vector $\varvec{\rho }^*$ but with the one computed from Istat data which is comparable up to a few fluctuation. This can be seen in Fig. 2a–c. While this means that the detailed balance is not exactly verified, the detailed balance condition is not used in the clustering and the population data of Istat is more accurate, thus ensuring that the computed currents are more representative of the real fluxes.

### Greedy modularity communities method (GMC)

This clustering algorithm is provided by the networkx Python library (greedy_modularity_communities). This algorithm, developed in^67^ and refined in^68,69^, relies on the optimization of the modularity *Q*. Let $W_{ij}$ be a weighted matrix, without self-loops, of the associated graph; for a given clustering *c*, the modularity is defined as^69^:

8 $$\begin{aligned} Q=\frac{1}{2m}\sum _{ij} W_{ij}- \frac{k_i k_j}{2m} \delta (c_i,c_j), \qquad \text {where} \qquad m=\frac{1}{2}\sum _{i,j} W_{ij} \qquad \text {and} \qquad k_i=\sum _j W_{ij} \end{aligned}$$

The quantity *m* generalises what would be the number of edges in a binary graph, $k_i$ is the generalised degree of the node *i*, and $c_i$ labels the cluster to which node *i* belongs.

To understand its meaning, consider the simpler case of an unweighted graph, where $W_{ij}=A_{ij}$ is the adjacency matrix. If connections are made at random but respecting the degrees $k_i$ and $k_j$ of the nodes *i* and *j*, then the probability of an existing link between these two nodes is $k_ik_j/2m$. This means that modularity measures the difference between the linkage of the node within a community cluster and what is expected from a random network. With increasing values of *Q*, one has an increasing deviation from a random choice of linkage. Also, looking at Eq. (8), we see that if there is only one cluster, then $\delta (c_i,c_j)\equiv 1$, and it is straightforward to see that in this case $Q=0$. In the opposite situation, where the clustering is made only of singletons then $\delta (c_i,c_j)=\delta _{ij}$; in this case as well, we see that $Q=0$. It is possible to show^70^ that, in between these extreme cases, there exists an optimal clustering corresponding to maximal modularity. The algorithm tests different levels of resolution through an agglomerative clustering method similar to the one presented in section “Spatial clustering”, aiming at finding the clustering of the network with maximal modularity.

### Effective distance matrix between nodes

Following ref^51^, we define the *effective distance* between two *adjacent* nodes *i* and *j* as $d_{ij}=1-\ln {\Pi _{ij}}$. If there exists a path going from *i* to *j* with *l* steps : $\Gamma _{ij}=\{(k_0=i,k_1),(k_1,k_2),\ldots ,(k_{l-1},k_{l}=j)\},$ the *direct length* $\lambda (\Gamma _{ij})$ of this path is the sum of the effective distances along its steps. We defined the *effective distance* $D_{ij}$ between *any* node as the minimal distance among all the existing paths from *i* to *j*:

9 $$\begin{aligned} \lambda (\Gamma _{ij})=\sum _{n=0}^{l-1} d_{k_n,k_{n+1}} ; \qquad \qquad D_{ij}=\min _{\Gamma _{ij}}\lambda (\Gamma _{ij}); \qquad \qquad \text {and} \qquad \qquad \Delta _{ij} =\left\{ \begin{array}{ll} 0 &{} \text{ if } \, i=j \\ d_{ij} &{} \text{ if } \, \Pi _{ij}\ne 0\\ d_{ji} &{} \text{ if } \, \Pi _{ij}=0 \, \text{ and }\, \Pi _{ji}\ne 0\\ D_{ij} &{} \text{ if } \, \exists \, \Gamma _{ij}\\ D_{ji} &{} \text{ if } \, \not \exists \, \Gamma _{ij} \, \text{ and }\, \exists \, \Gamma _{ji} \\ +\infty &{} \text{ elsewhere. } \end{array} \right. \end{aligned}$$

The *effective distance matrix* used in CVS is $\Delta ^S=(\Delta +\Delta ^t)/2$ the symetric part of $\Delta$.

This definition is valid for any weighted directed graph. In particular, the last line is not needed if the graph is weakly connected ($\forall (i,j),\, \exists \, \Gamma _{ij}\, \text {or}\, \exists \, \Gamma _{ji}$). Similarly, the two last lines are not needed if it is strongly connected ($\forall (i,j),\, \exists \, \Gamma _{ij}$).

In our case, the most representative transition matrix of the non-confined period, $\overline{\Pi }^{C_0}$ is strongly connected while $\overline{\Pi }^{C_1}$, the graph associated with the most representative transition matrix for the confinement period is not even weakly connected, and its connected components are not always strongly connected. We add that, on a computer, ‘infinite’ must be represented as a large number; this value was defined as 100 times the maximum of the well-defined elements of $\Delta$. The effective distance matrix is normalized by its mean value: $\Delta ^S \leftarrow \Delta ^S / \overline{\Delta ^S}$ where $\overline{\Delta ^S}=\frac{1}{N^2}\sum ^N_{i,j} \Delta ^S_{ij}$. In this way, the agglomerative clustering operations on the distance matrix do not depend on the large-scale cutoff.

#### Critical variable selection method (CVS)

**Figure 4:**
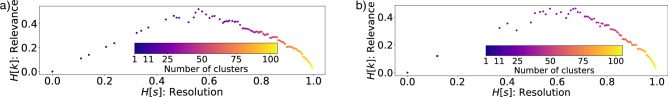
CVS method: Right panels: Relevance versus resolution for the *N* clusterings obtained by hierarchical clustering of: (**a**) the most representative matrix of temporal cluster $C_0$ (confined, top);(**b**) $C_1$ (unconfined, bottom). For both case the clustering with the highest relevance defined the optimal clustering.

The critical variable selection method, also known as resolution-relevance^71–76^, has been successful in identifying optimal clustering for the reduction of complexity in the representation of biomolecules^77^ or for a protein conformational landscape^78^.

Considering a set of *N* objects and a given clustering of them, we labeled the *K* clusters by $s\in \llbracket 1, K \rrbracket$ and defined $k_s$ to be the number of objects in cluster *s*. $k_s/N$ is the empirical probability for an object to belong to cluster *s*.

10 $$\begin{aligned} \text {The }{} \textit{resolution} \; \text {is defined as the Shannon entropy of this probability distribution:} \qquad H[s]= -\sum ^{K}_{s=1} \frac{k_s}{N} \log _N{\frac{k_s}{N}} \end{aligned}$$

where $\log _N$ is the logarithm in base *N* such that $\log _N N = 1$. $H[s] = 0$ when all objects belong to only one cluster, and $H[s]=1$ at the other extreme, when each object has its own separate cluster.

Resolution alone, however, is not sufficient to identify an optimal level of informativeness of a given clustering. A second quantity, the *relevance* is defined based on the number of clusters containing *k* objects, $m_k$^45^:

11 $$\begin{aligned} m_k=\sum ^{K}_{s=1}\delta _{k,k_s}. \qquad \text { The } \; {relevance} \; \text { is defined as follows:} \qquad H[k]=-\sum ^{N}_{k=1} \frac{km_k}{N} \log _N{\frac{km_k}{N}}. \end{aligned}$$

In the latter expression, the factor $\frac{km_k}{N}$ is the empirical probability that a randomly chosen object in the collection belongs to the cluster with *k* elements in it. The relevance is the Shannon entropy associated with this second empirical probability. For both limit cases of 1 and *N* clusters, $H [k] = 0$, the relevance being non-negative otherwise^45,78^. The maximum relevance thus corresponds to an optimal clustering, i.e. to the most informative partition of the collection of objects.

We then performed an agglomerative clustering of the nodes representing provinces using the distance introduced above and computed for each number of clusters from 1 to *N* the corresponding values of resolution and relevance (see Fig. 4 right panels). The optimal partition of provinces was defined as the clustering with the maximum relevance value.

#### Variation of information (VI), a measure for cluster similarity

In order to quantify the similarity of the clustering, we measure the *Variation of Information* (VI). The *Variation of Information* is defined as^53^ $VI[C,C']=2H[C,C']-H[C]-H[C'],$ where *C* and $C'$ are two partitions of a set of *N* object containing respectively *K* and $K'$ clusters. $H[C,C']$ the *cross entropy* between the two partitions, and *H*[*C*] the Shannon entropy (or resolution) of clustering *C*, are defined as:

$$H[C,C']=\sum ^K_{i=1}\sum ^{K'}_{j=1} \frac{n_{ij}}{N} \log \left(\frac{n_{ij}}{N}\right), \hspace{0.2cm} \text {with:} \hspace{0.3cm} n_{ij}=|C_i \cap C'_j|,\;\; (i,j) \in \llbracket 1,K \rrbracket \times \llbracket 1,K' \rrbracket ; \hspace{0.3cm} \text {and} \hspace{0.3cm} H[C]= -\sum ^{K}_{k=1} \frac{k_s}{N} \log {\frac{k_s}{N}}$$

with $k_s=|C_s|$ the number of objects in cluster *s*. $VI[C,C']$ ranges from 0 to *log*(*N*). *VI* is also related to *the mutual Information*, $I[C,C']$, share by two partitionings as explained in detail in ref^53,54^.

### Supplementary Information

Supplementary Information.

## Data Availability

All the derivative datasets generated and analysed during this study are included in this published article in Clustering_Meta_matrices.zip. The Facebook mobility datasets are provided under an academic license agreement with Meta in the context of the “Meta Data for Good” program, through which data are released by Meta upon request to non-profit organizations and academics, see dataforgood.facebook.com. The Sars-Cov2 provincial Italian data set comes from the ISS^79^ (Italian National Institute of Health), and the official census of provincial populations from Istat^50^ (Italian National Institute of Statistics) and are publicly available data sets.
