# Supplementary material for: Quality assessment and community detection methods for anonymized mobility data in the Italian Covid context
Source: Sci Rep. 2024 Feb 26;14:4636. doi: 10.1038/s41598-024-54878-0 (PMC10897296; doi:10.1038/s41598-024-54878-0)
Supplement: Supplementary file 1 — Supplementary Information. [file 41598_2024_54878_MOESM1_ESM.pdf]

Supplementary Materials

Jules Morand,* Shoichi Yip, Yannis Velegrakis, Gianluca Lattanzi, Raffaello Potestio, and Luca Tubiana
*Physics Department, University of Trento, via Sommarive, 14 I-38123 Trento, Italy, INFN-TIFPA,
Trento Institute for Fundamental Physics and Applications, I-38123 Trento, Italy and
Information and Computing Science, University of Trento, Italy and Utrecht University, Netherlands*

Appendix A: List of Provinces used in the study

node index	Provinces name	Car plate code	ITTER107 code	Population 01 Jan 2020
0	Agrigento	AG	ITG14	412427.0
1	Alessandria	AL	ITC18	407049.0
2	Ancona	AN	ITE32	461745.0
3	Aosta	AO	ITC20	123337.0
4	Ascoli Piceno	AP	ITE34	202317.0
5	L'Aquila	AQ	ITF11	288439.0
6	Arezzo	AR	ITE18	334634.0
7	Asti	AT	ITC17	207939.0
8	Avellino	AV	ITF34	399623.0
9	Bari	BA	ITF42	1224756.0
10	Bergamo	BG	ITC46	1102670.0
11	Biella	BI	ITC13	169560.0
12	Belluno	BL	ITD33	198518.0
13	Benevento	BN	ITF32	263460.0
14	Bologna	BO	ITD55	1015701.0
15	Brindisi	BR	ITF44	379851.0
16	Brescia	BS	ITC47	1254322.0
17	Barletta-Andria-Trani	BT	IT110	379251.0
18	Bolzano	BZ	ITD10	535774.0
19	Cagliari—Sud Sardegna	CA	ITG27—IT111	754878.0
20	Campobasso	CB	ITF22	210599.0
21	Caserta	CE	ITF31	900293.0
22	Chieti	CH	ITF14	372473.0
23	Caltanissetta	CL	ITG15	250550.0
24	Cuneo	CN	ITC16	580789.0
25	Como	CO	ITC42	594657.0
26	Cremona	CR	ITC4A	351287.0
27	Cosenza	CS	ITF61	671171.0
28	Catania	CT	ITG17	1068835.0
29	Catanzaro	CZ	ITF63	341991.0
30	Enna	EN	ITG16	155982.0
31	Forlì-Cesena	FC	ITD58	391524.0
32	Ferrara	FE	ITD56	340755.0
33	Foggia	FG	ITF41	597902.0
34	Firenze	FI	ITE14	994717.0
35	Fermo	FM	IT109	168485.0
36	Frosinone	FR	ITE45	468438.0
37	Genova	GE	ITC33	816250.0
38	Gorizia	GO	ITD43	138666.0
39	Grosseto	GR	ITE1A	216989.0
40	Imperia	IM	ITC31	208561.0
41	Isernia	IS	ITF21	80170.0
42	Crotone	KR	ITF62	161744.0
43	Lecco	LC	ITC43	332435.0
44	Lecce	LE	ITF45	772276.0
45	Livorno	LI	ITE16	326716.0
46	Lodi	LO	ITC49	227064.0
47	Latina	LT	ITE44	565840.0
48	Lucca	LU	ITE12	381890.0
49	Monza e Brianza	MB	IT108	870112.0
50	Macerata	MC	ITE33	305249.0
51	Messina	ME	ITG13	599990.0
52	Milano	MI	ITC45	3237101.0
53	Mantova	MN	ITC4B	404440.0
54	Modena	MO	ITD54	702787.0
55	Massa-Carrara	MS	ITE11	188395.0
56	Matera	MT	ITF52	191663.0
57	Napoli	NA	ITF33	2967117.0
58	Novara	NO	ITC15	361845.0
59	Nuoro—Ogliastra	NU	ITG26	199349.0
60	Oristano	OR	ITG28	150812.0
61	Palermo	PA	ITG12	1199626.0
62	Piacenza	PC	ITD51	283889.0
63	Padova	PD	ITD36	930898.0
64	Pescara	PE	ITF13	313346.0
65	Perugia	PG	ITE21	641318.0
66	Pisa	PI	ITE17	417245.0
67	Pordenone	PN	ITD41	310158.0
68	Prato	PO	ITE15	264397.0
69	Parma	PR	ITD52	450044.0
70	Pistoia	PT	ITE13	289256.0
71	Pesaro e Urbino	PU	ITE31	351993.0
72	Pavia	PV	ITC48	534691.0
73	Potenza	PZ	ITF51	348336.0
74	Ravenna	RA	ITD57	386007.0
75	Reggio di Calabria	RC	ITF65	518978.0
76	Reggio nell'Emilia	RE	ITD53	524193.0
77	Ragusa	RG	ITG18	315082.0
78	Rieti	RI	ITE42	150689.0
79	Roma	RM	ITE43	4222631.0
80	Rimini	RN	ITD59	336916.0
81	Rovigo	RO	ITD37	229097.0
82	Salerno	SA	ITF35	1060188.0
83	Siena	SI	ITE19	262046.0
84	Sondrio	SO	ITC44	178208.0
85	La Spezia	SP	ITC34	214879.0
86	Siracusa	SR	ITG19	383743.0
87	Sassari—Olbia-Tempio	SS	ITG25	474142.0
88	Savona	SV	ITC32	267748.0
89	Taranto	TA	ITF43	558130.0
90	Teramo	TE	ITF12	299402.0
91	Trento	TN	ITD20	542158.0
92	Torino	TO	ITC11	2205104.0
93	Trapani	TP	ITG11	415233.0
94	Terni	TR	ITE22	218254.0
95	Trieste	TS	ITD44	230623.0
96	Treviso	TV	ITD34	876755.0
97	Udine	UD	ITD42	517848.0
98	Varese	VA	ITC41	878059.0
99	Verbano-Cusio-Ossola	VB	ITC14	154233.0
100	Vercelli	VC	ITC12	165760.0
101	Venezia	VE	ITD35	839396.0
102	Vicenza	VI	ITD32	852861.0
103	Verona	VR	ITD31	927108.0
104	Viterbo	VT	ITE41	307592.0
105	Vibo Valentia	VV	ITF64	150702.0

* jules.morand@unitn.it

8 **Appendix B: Regions of Italy**

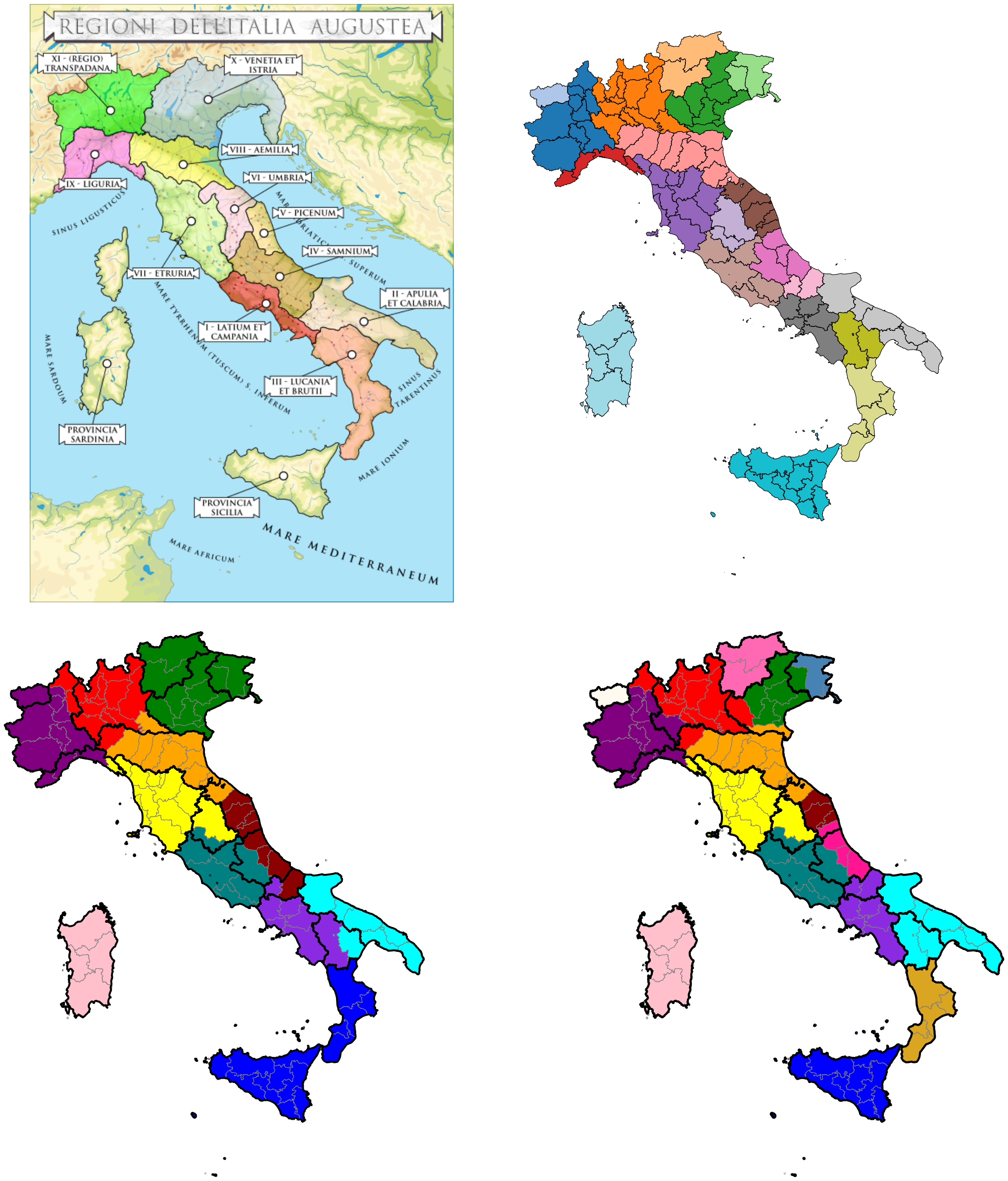

FIG. SI.1. Top left: The proposed partition of Emperor Augustus
Source: https://it.wikipedia.org/wiki/Regioni_dell%27Italia_augustea#/media/File:Regioni_dell'Italia_Augustea.svg.
Top right: Administrative borders of the provinces of Italy considered in the study (black lines) and of the regions (in colors) as there are defined now. Bottom: Both optimal spatial clusterings for the non-confided periods (left with GMC, right with CVS).

Although an in depth study going well beyond the purpose of the present manuscript is certainly necessary to ascertain the origin and relevance of this agreement, it is interesting to observe that the 11 clusters found through modularity also appear to qualitative resemble those reported by Pliny the Elder (References: Cifani, 2010,Pliny the Elder,Naturalis Historia III. 46, Briet, Philippe).

Appendix C: About Perron-Frobenius (PF) theorem for stochastic matrix

In the graph identified by the mean matrix $\overline{\Pi}$ there is a non-zero probability to reach any node from any other node in a finite number of steps, that is, the graph is strongly connected and aperiodic. (To say a graph is aperiodic is equivalent to saying that its representative matrix is irreducible or saying that the random walk on the graph is ergodic.) Then, the transition matrix representing the graph is non-negative and irreducible.

For a general non-negative irreducible matrix, $\mathbf{\Pi}$, the PF theorem then ensures that the highest eigenvalue λ^* of $\mathbf{\Pi}$ is not degenerate. It is often called the PF eigenvalue and we name PF left eigenvector l^* (and right eigenvector r^*) its associated eigenvectors.

A consequence of the theorem in this case is also that any distribution on which we apply the matrix successively, will concentrate, in the long time (long path limit), to the stationary density vector $\rho^*_i = l^*_i.r^*_i$.

We explain it here in our specific case where the matrix is stochastic.

The normalization of $\mathbf{\Pi}$ is such that it is stochastic, i.e. each its raws sum to 1, i.e 1 is eigenvalue of $\overline{\Pi}$ and is associated with the right eigenvector $\boldsymbol{r}^* = \mathbf{1}_{np} = (1, 1, \ldots, 1)^T$ and $\boldsymbol{l}^*$:

$$\boldsymbol{l}^* = \boldsymbol{l}^*\overline{\Pi} \tag{C1}$$

Moreover, one can also show that in this case, 1 is the maximum eigenvalue possible and the PF theorem ensure it is unique, as well as its associated eigenvectors.

Therefore, for stochastic matrix, we commonly identify $\boldsymbol{\rho}^* = \boldsymbol{l}^*$ as the stationary density vector but in general, r^*_i may be something else than the $\mathbf{1}$ and the PF eigenvalue different than 1.

In the following, we show the long-time limit convergence in our case.

Because the PF left eigenvector of $\mathbf{\Pi}$ is the unique invariant, it ensures the detail balance of the associated Markov process:

$$\rho^*_i \Pi_{ij} = \Pi_{ji}\rho^*_j$$

. Multiplying the left and the right by $\rho_i^{*-1/2}$ and $\rho_j^{*-1/2}$, we obtain that

$$\rho_i^{*1/2}\Pi_{ij}\rho_j^{*-1/2} = \rho_i^{*-1/2}\Pi_{ji}\rho_j^{*1/2} = (\rho_j^{*-1/2}\Pi_{ij}\rho_i^{*1/2})^T. \tag{C2}$$

Hence the matrix S defined by the elements

$$S_{ij} = \rho_i^{*1/2}\Pi_{ij}\rho_j^{*-1/2}, \tag{C3}$$

is equal to its own transposed, and so is symmetric.
Defining the transformation $U = diag(\rho_i^{*1/2})$ we have:

$$S = U^{-1}\mathbf{\Pi}U, \tag{C4}$$

then S have the same eigenvalues (for S, left and right eigenvector are the same) and is symmetric, so diagonalizable in real space, so Π itself is diagonalizable.

So there exists a mapping, i.e. a base of the vector space, O such that :

$$\Pi = O^{-1}\mathbf{\Lambda}O$$

with $\mathbf{\Lambda} = diag(\lambda^*, \lambda_1, ..., \lambda_N)$.

The PF theorem ensures that for our stochastic matrix $\lambda^* = 1 > |\lambda_i| \geq 0$, $i = 1, .., N$,
Hence, in the long time limit (or long path limit).

$$\mathbf{\Lambda}^t \sim diag(1, 0, 0, 0, ..., 0)$$

The principal (or Perron-Frobenius) eigenvalue $\lambda^* = 1$ will dominate and any non-trivial distribution $\boldsymbol{\rho}$ over the nodes will converge to the unique stationary density vector:

$$\boldsymbol{\rho}\mathbf{\Pi}^t \sim \boldsymbol{\rho}^*.$$

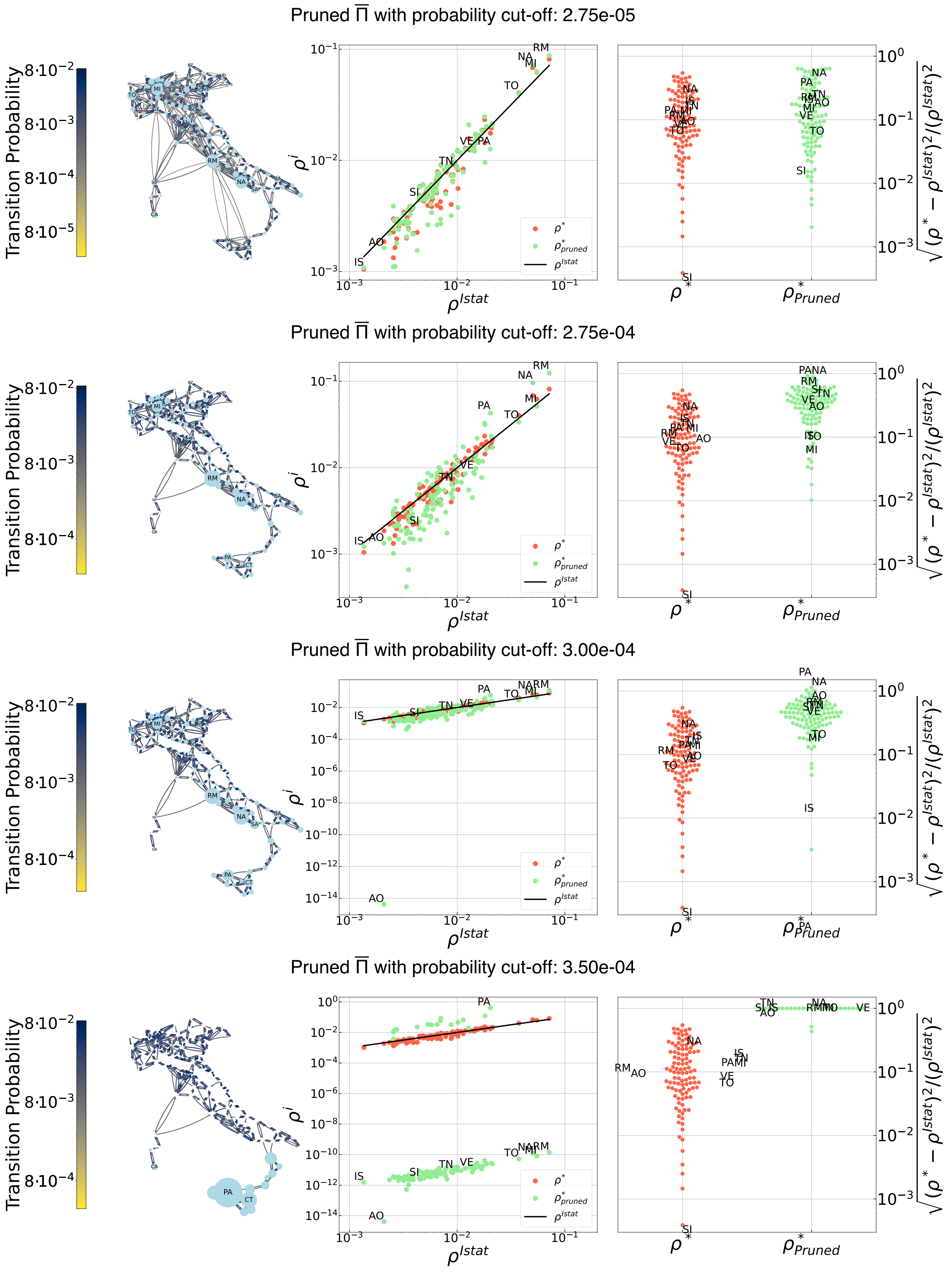

FIG. SI.2. Perron Frobenius theorem checked by pruning the links of the mean network of Italian mobility. We see that the importance of small link to predict the population vector. On the top panel, the full Network of the mean matrix $\overline{\Pi}$ is represented with all the links (self-loops excluded). The smaller links are progressively suppressed, with increasing cutoff $(2,75.10^{-5}, 2,74.10^{-4}, 3.10^{-4}, 3,5.10^{-4})$, and the prunned matrix is normalised to be a stochastic matrix. The Perron Fobenius first left eigenvectors of each pruning ρ^* is then compute and shown on the second colomn versus ρ^{Istat} the Istat data. The right most column is the standard deviation with this vector: we see a global increasing of these deviation from the Italian State data. For higher cuttoff, the graph becomes only weakly connected provicking some provinces to be 'sink' of the random walk.

Appendix D: Transition matrices time series

We display here more information contained in the time series of weekly-averaged daily transition matrices. First, the temporal clustering process present in Material and Methods section 3.3 can be represented in a tree (dendrogram) in which the child branches at each step represent the pairs of clusters that merge into a parent branch. We report this hierarchical clustering dendrogram in Fig. ??. The length of the branches (y-axis) corresponds to the cophenetic

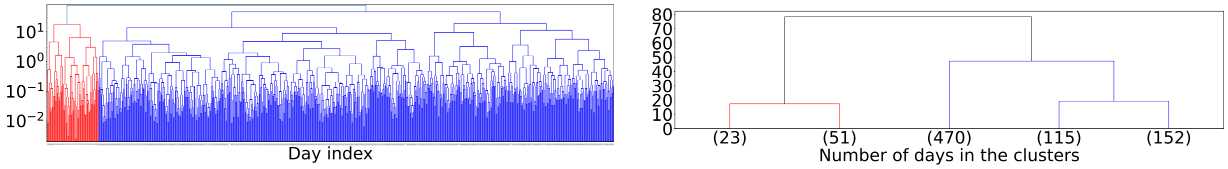

FIG. SI.3. Hierarchical clustering dendrogram of the day-by-day transition matrices: Left: full dendrogram, Right: dendrogram cut at the level of 5 clusters with in the x-axis the number of days of each clusters.

distance, a distance which measures the level of similarity between two merged clusters. Fig. ??a) displays the full dendrogram from individual nodes to one unique cluster. On Fig. ??b this dendrogram is cut at the level of 5 clusters, after which the cophenetic distance increases significantly; the numbers in parentheses (in the x-axis) are the number of nodes belonging to each cluster.

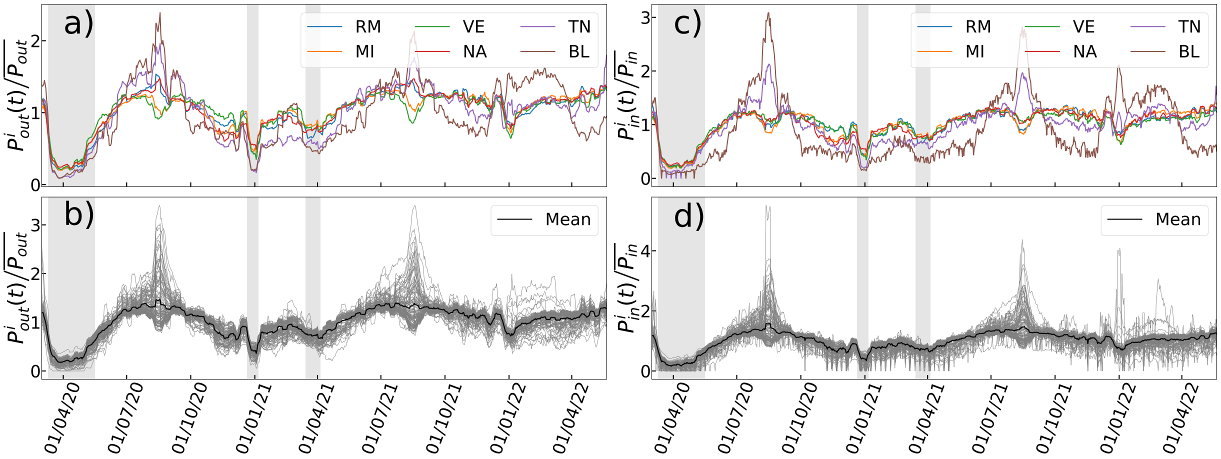

FIG. SI.4. a) and b) Probability of going out of the province versus time a) for i =BL, MI, NA, RM, TN, and VE and b) for the mean over the province in black and the whole distribution of grey. c) and d) Probability of going in the province versus time c) for i =BL, MI, NA, RM, TN, and VE and d) for the mean over the province in black and the whole distribution of grey. All probability distributions have been plotted and re-scaled by their temporal average to obtain a collapse of the curves. Gray shaded areas represent national lockdown periods.

The movement pattern of single provinces can be brought to collapse on two master curves with an appropriate rescaling, see Fig. ??a)-d). Specifically, this can be done by considering the normalized probability to move out of a province,shown in Fig. ??a), b)

$$\frac{P^i_{out}(t)}{\overline{P_{out}}} = \frac{1-\Pi_{ii}(t)}{1-\overline{\Pi}_{ii}}, \text{ and the normalized probability to move into a province } \frac{P^i_{in}(t)}{\overline{P_{in}}} = \frac{\sum_{j\neq i}\Pi_{ji}(t)}{\sum_{j\neq i}\overline{\Pi}_{ji}}, \tag{D1}$$

reported in Fig. ??c), d). As can be seen from panels c), e) all provinces display a similar behavior in these two quantities, and the first two lockdowns become apparent as periods of low mobility. The Z-score, i.e. the time average of the fluctuation of $P^i_{in}(t)$ and $P^i_{out}(t)$ with respect to the mean over provinces, is defined and displayed in supporting information (Fig.S5).

Interestingly, we also note that some provinces show a large deviation in both quantities in correspondence of summer and winter months. To rationalize this, we look at the provinces showing peaks of mobility in those periods, and found them to correspond with those having a high touristic vocation, as for example Belluno (BL) and Trento (TN), near the Dolomites, and Sicilian provinces, see Fig. ??a), b). While at first the fact that Rome and Venice (VE)

do not show these peaks might be unexpected, we recall that our data only follow the movement of Italian citizens, and that during Covid there was a strong push to take holidays outside of cities.

Appendix E: In and outgoing probability for each spatial cluster

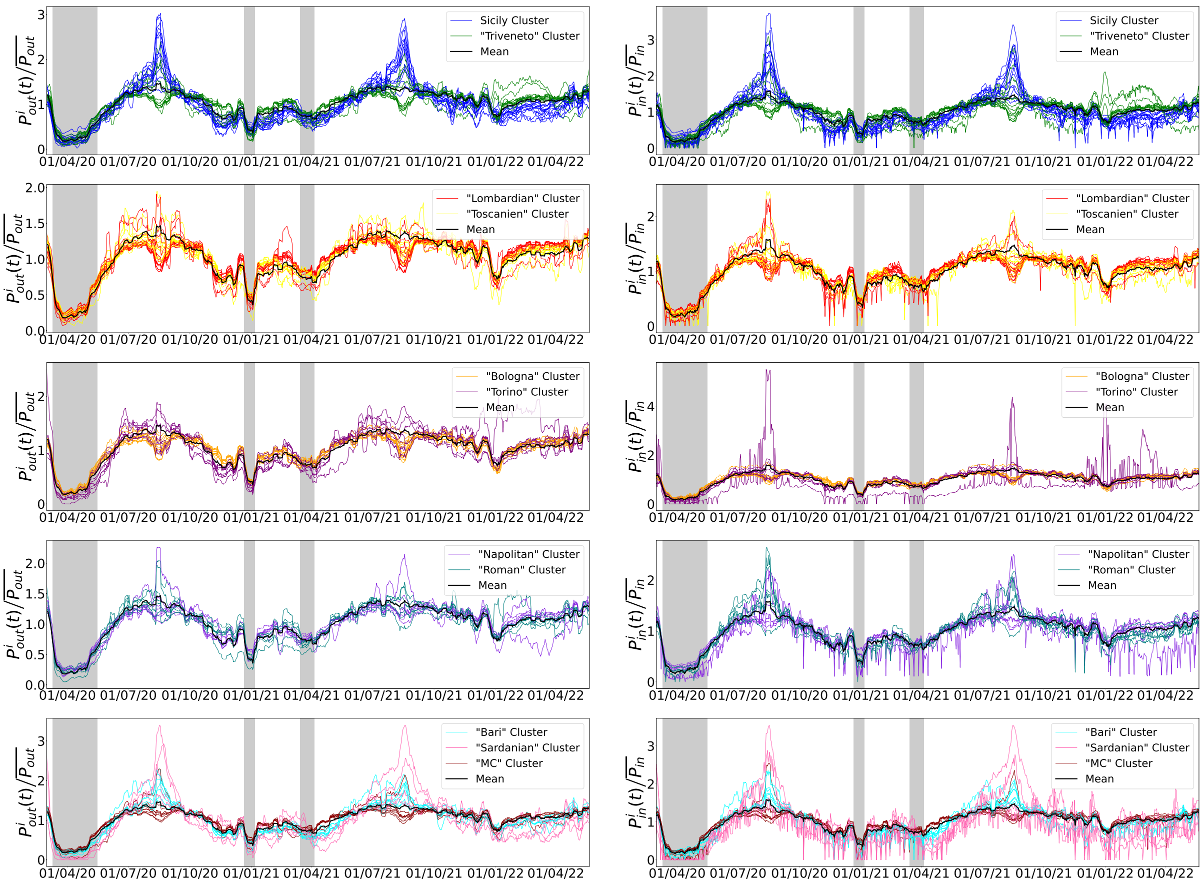

FIG. SI.5. Left Panels :Outgoing probability for each cluster, the colors correspond to the clusters of the non-confined case using GMC. Right Panel: Ingoing probability for each cluster the colors correspond to the clusters of the non-confined case using GMC .

Appendix F: Z-score of the probability of going in and out of provinces

To better see which provinces differ the most from the mean trend we compute the Z-score, which is defined for the time series $X_i(t)$ of province i as:

$$Z_i = \frac{1}{T}\sum_{t=0}^{T}\frac{|X_i(t)-\mu(t)|}{\sigma(t)} \tag{F1}$$

with $\mu(t) = \langle X_i(t)\rangle$ being the average over provinces and $\sigma(t) = \sqrt{\langle X_i(t)-\mu(t)\rangle}$ the standard deviation at time t.

In Fig. **??**, we show at the top a map with the Z score for the outgoing probabilities, and on the bottom one for ingoing probabilities for each province.

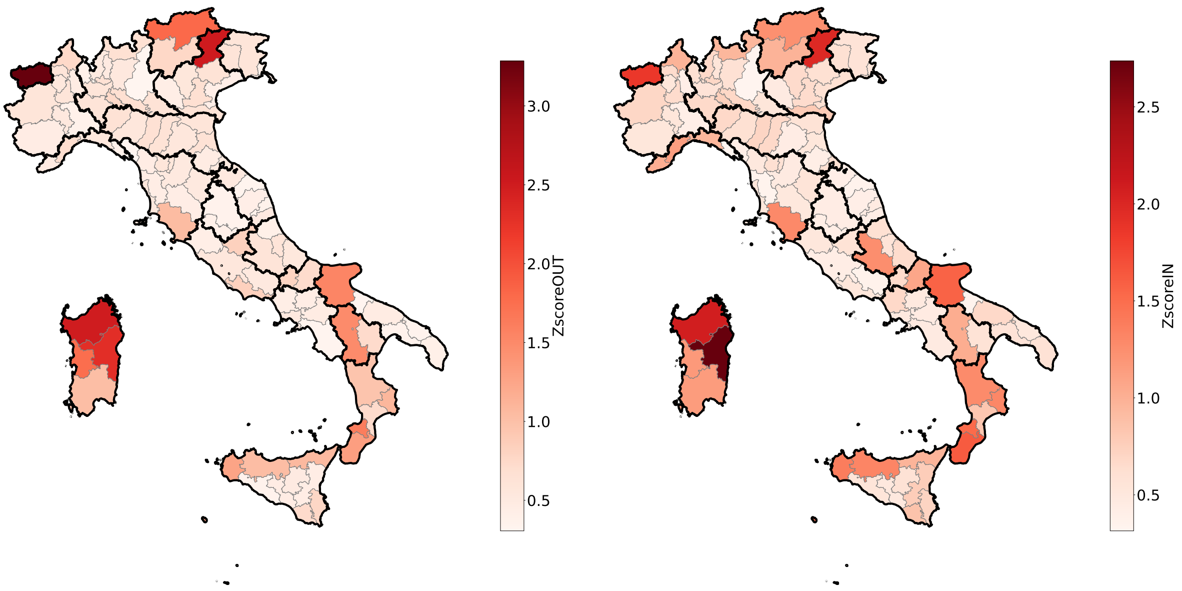

FIG. SI.6. Map of the 2-year average Z score by province. The Z scores defined Eq. **??** are the average fluctuations by provinces of the probability of moving in (Z score IN) and out (Z score OUT) of the province.

Appendix G: Clustering of the mean current Matrix

In Fig.**??**, on the top is displayed a representation of the mean current matrix sorted by clusters and by weight, one sees that the method is satisfactory giving well-defined blocks corresponding to each community. On the bottom panel, we see that the clusters correspond, apart from very few border cases,(and Umbria is split apart) to a group of regions in Italy. In detail, for the ten clusters found, we have:

- The green cluster corresponds perfectly to the "Triveneto" region (that is, Veneto, Friuli-Venezia-Giulia, and the provinces of Trento and Bolzano).
- The red one to Lombardia with the exception of Mantova plus the two provinces of Verbano/Cusio/Ossola and Novara (belonging to Piemonte) and Piacenza (belonging to Emilia-Romagna).
- The dark purple corresponds to the region of Valle d'Aosta and Piemonte (minus VB and NO) and Liguria, at the exception of Spezia.
- The yellow cluster is Toscana plus the provinces of Spezia (Liguria) and Perugia (Umbria)
- The orange one corresponds to the region of Emilia-Romagna at the exception of Piacenza (PC) and adds the provinces of Pesaro/Urbino (Marche) and Mantova (Lombardia).
- The grey cluster is the regions of Marche (minus PU), Abruzzo (minus AQ) and Molise (minus IS)
- The teal one matches with the regions of Lazio and Sardegna (plus TE (Umbria) and AQ (Abruzzo))
- The light purple corresponds to the region of Campania plus the province of Isernia belonging to Molise.
- The light blue cluster corresponds perfectly o the regions of Puglia and Basilicata
- Finally, the blue cluster perfectly to the regions of Calabria and Sicilia.

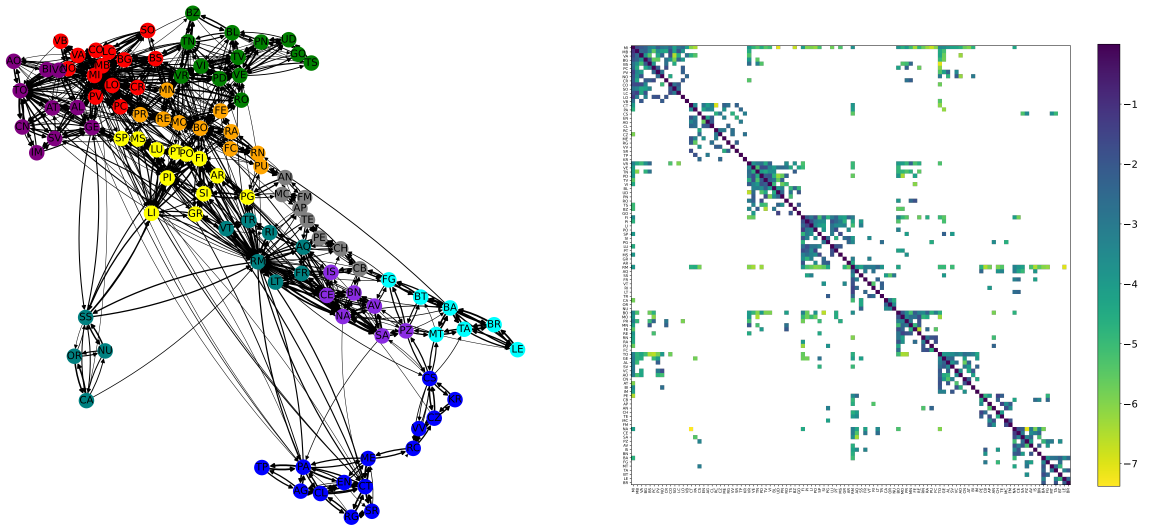

FIG. SI.7. Italian provinces clustered using GMC by communities using the all time-averaged matrix.Left panel: Graph representation of the community clustering with colors corresponding to the different clusters, the widths of the links are proportional to the logarithm of the transition probability. At the exception of Sardinia that is in Rome cluster here, the clustering is the same than non-confined most representative matrix using GMC. Right panel: Representation of the clustered mean current matrix, for visualization the shades are in log10 of the mean probabilities of going from one province to another.

Appendix H: Network representation of the spatial partition with the two clustering methods

We display here the full network visualization of the two most representatives obtained for the two temporal clusters, the widths of the links are proportional to the logarithm of the transition probability.

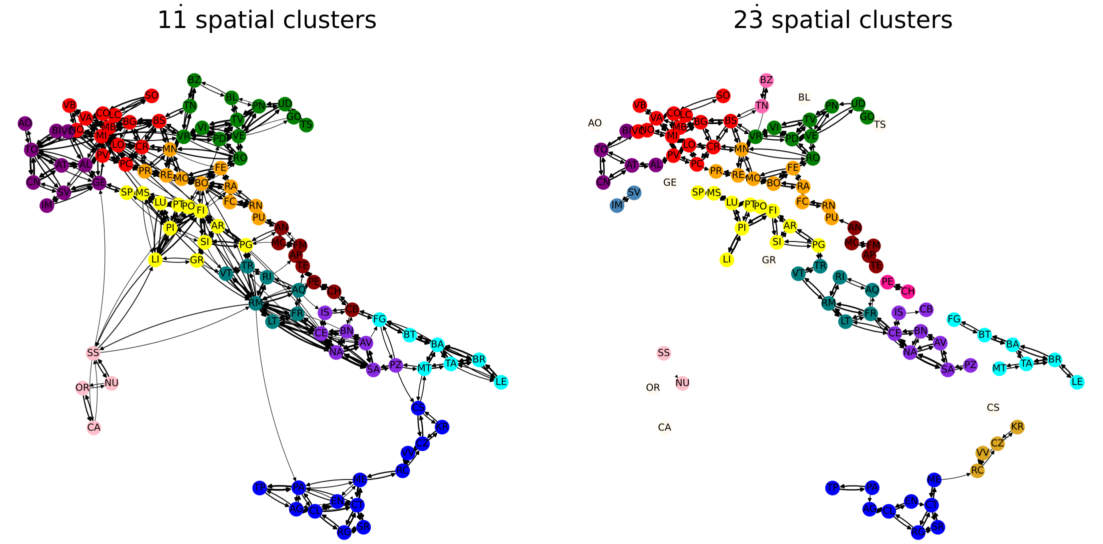

FIG. SI.8. Directed graph representation of the most representative matrices for non-confined cluster C_0 (left panel) and confined one C_1 (right panel) the optimal clustering in using the greedy modularity method.

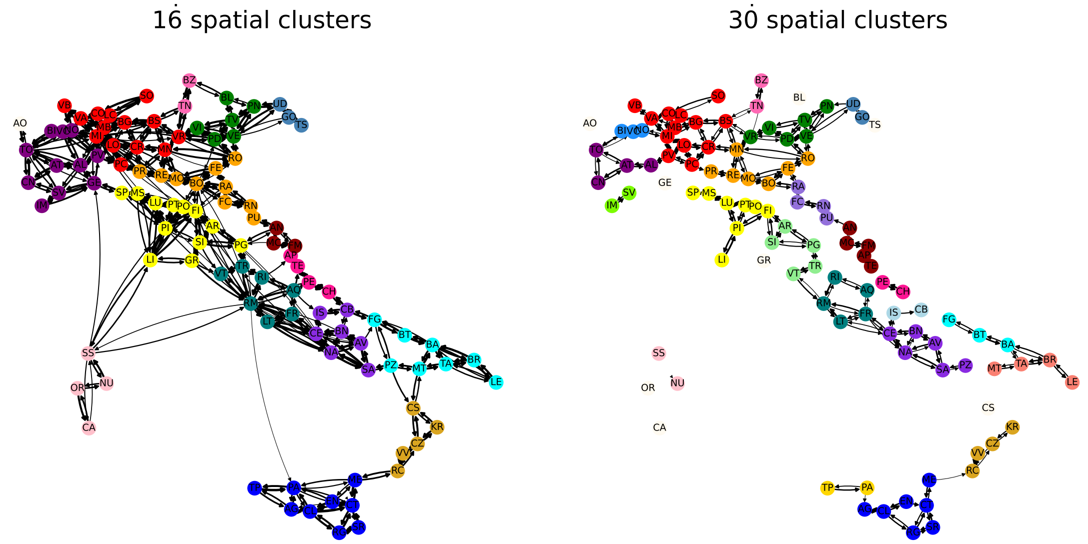

FIG. SI.9. Directed graph representation of the most representative matrices for non-confined cluster C_0 (left panel) and confined one C_1 (right panel) the optimal clustering in using the critical variable selection method.

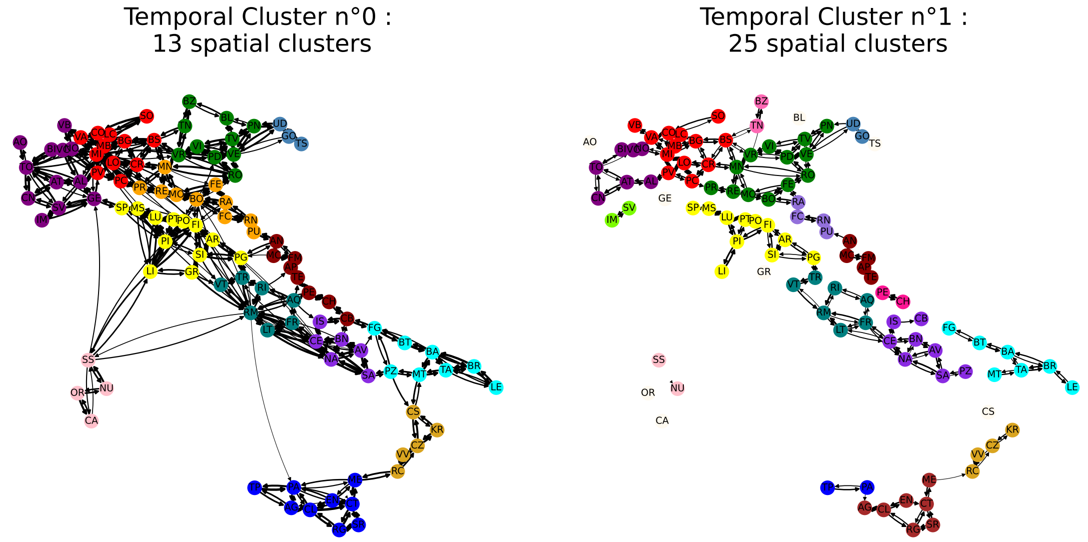

FIG. SI.10. Directed graph representation of the most representative matrices for non-confined cluster C_0 (left panel) and confined one C_1 (right panel) the optimal clustering in using the greedy modularity, but this time apply to the most representative transition probability matrices.

Appendix I: Data Preparation

1. Data Sources

Mainly two data sources were used for our analysis: the **Facebook Data for Good Italy Coronavirus Disease Prevention Map** data and the **COVID-19 data** published by the ISS (Istituto Superiore di Sanità - National Institute of Health) - INFN (Istituto Nazionale di Fisica Nucleare - National Institute for Nuclear Physics) collaboration. Respectively, the two data sources contained the following datasets that were of interest to us:

- The **Movements Between Administrative Regions** dataset describes the number of Facebook users that

move between two NUTS-3 administrative regions (aka *province*). The temporal aggregation of the dataset is of 8 hours, meaning that if a person is checked-in in region A in a certain time frame, and the same person is found to be checked-in in another region B in the subsequent time frame, then a movement between regions A and B are counted. A 24-hour the day is divided into three time frames: 00:00-08:00, 08:00-16:00 and 16:00-24:00. Two types of data are considered: the baseline, which is computed by taking the average on the same weekday for the same weekdays, and the people during the crisis, which is the actual number of people detected in the specified DateTime. Only users of the Facebook app that have the Location History option enabled are counted, and also if the aggregation yields counts under 10 units then the datum is discarded.

- The **New Positive Cases By Date** dataset describes the number of new positive SARS-CoV-2 cases, aggregated by date and province of detection. This dataset does not suffer from the lag between detection and publication, unlike the Dipartimento della Protezione Civile (Department for Civil Defense) data. The number of cases is the result of a window average over a week, where the final result is the day in the middle of the week (the fourth day of the week).

2. The choice of the stack

In order to perform the extraction, loading, and transformation of the data various paths have been explored, but our choice fell on the current technological stack.

- **Python** is the main scripting language and piece of software used throughout the whole pipeline. Its user-friendliness, its widespread use among both the industry and researchers, and the availability of great libraries for data science and visualization made it our natural choice for our purposes. In particular, the libraries mainly used by us are Selenium (web browser automation) and Pandas (data analysis and manipulation).

- **Miller** is a toolkit for data munging. It allows quick CSV manipulations and it contains several powerful commands, that can also be chained one after the other.

- **Bash** is used for integrating and preprocessing various data sources. It is extremely flexible and compatible with most of Unix-like systems, and for certain types of data science workloads it can quickly and efficiently get the work done.

- **DuckDB** is an embeddable analytical database. It is similar to SQLite, in that the database system runs within a host process, but it is optimized for analytical (OLAP) workloads. It allows for manipulations *à la* Pandas but also has full-query optimization and transactional storage. It is a good choice for our purposes since it allows faster queries to be made, it does not require the maintenance of a DB stack and it integrates very well with Python thanks to the DuckDB Python API.

3. Gathering the data

The **Facebook Data for Good** data can only be downloaded by using an online interface, and each transaction is size-capped (i.e. Movements Between Administrative Regions data for more than a two-weeks period would not be downloaded). In order to facilitate and speed up the sourcing of the data, a bulk download tool was developed. The tool makes use of Selenium, a Python library for browser automation, that allows automated workflows that simulate human interaction with a browser. The resulting raw data are available as zip archives containing many CSV files.
The **COVID-19 data** by ISS/INFN is published as a single zip archive containing many CSV files, one for each aggregation, data type and province/region.
Reference tables are also essential for the analysis, as they allow data integration between incoherent definitions (in particular, concerning spatial aggregation units) between different datasets. Some of them are manually compiled, and others are aggregate data extracted from the original datasets:

- The **provinces** identifications conversion table has been created manually. It contains the correspondence between IDs for provinces in the Facebook dataset, the ISS/INFN dataset, and the "car number plate code" two letters characters).

- The **locations** reference table contains the latitude and longitude for each province.

4. Cleaning and loading the data

The raw data is then loaded into our database for further analysis.

First of all, the archives are unpacked and the data is cleaned for our purposes with the use of Miller. The following operations are performed:

- The data is filtered in order to get only data for Italian provinces (generally Facebook uses rectangular bounding boxes to get subsets of data).
- Minor changes in data formats are operated (such as missing date-time imputation and format correction for date-time strings).
- Null entries are discarded.
- Only columns of our interest are selected.

Then the data are piped through an SQL COPY command, that loads it in a DuckDB database.

5. Transforming the data

This is the last step of our data preparation pipeline.

In this step, we transform the raw data contained in the database into SQL tables with SQL views, in such a way that they can be accessed easily and are expressed in a manner that is optimal for the analysis purposes of our research.

The Movements Between Administrative Regions dataset has rows aggregated and summed over with the new definitions of provinces as defined in the reference table.

Starting from this table, then multiple views are created:

- total number of people moving from each origin place by date;
- total number of people moving between places summed over by each day;
- daily probability that a movement between places happens (aka *transition matrix*);
- total probability that a movement between places happens;
- weekly rolling average of the daily probability of movement between places.

The COVID-19 ISS row dataset is also aggregated and summed over the province using the new definitions as defined in the reference table.**??**.
